# Interactions between RASA2, CADM1, HIF1AN gene polymorphisms and body fatness with breast cancer: a population-based case–control study in China

**DOI:** 10.18632/oncotarget.21530

**Published:** 2017-10-05

**Authors:** Zheng Zhu, Zhimei Teng, Fränzel J.B. van Duijnhoven, Meihua Dong, Yun Qian, Hao Yu, Jie Yang, Renqiang Han, Jian Su, Wencong Du, Xingyu Huang, Jinyi Zhou, Xiaojin Yu, Ellen Kampman, Ming Wu

**Affiliations:** ^1^ Department of Chronic Disease Control, Jiangsu Provincial Center for Disease Control and Prevention, Nanjing, Jiangsu, China; ^2^ Division of Human Nutrition, Wageningen University, Wageningen, The Netherlands; ^3^ Department of Chronic Disease Control, Wuxi Center for Disease Control and Prevention, Wuxi, Jiangsu, China; ^4^ School of Public Health, Southeast University, Nanjing, Jiangsu, China

**Keywords:** breast cancer, RASA2, CADM1, HIF1AN, gene polymorphisms

## Abstract

Genome-wide association studies (GWAS) have indicated that gene polymorphisms in alleles of RAS p21 protein activator 2 (RASA2), cell adhesion molecule 1 (CADM1) and hypoxia inducible factor 1 alpha subunit inhibitor (HIF1AN) are associated with the risk of obesity. In this study, we explored the interactions between candidate SNPs of RASA2 (rs16851483), CADM1 (rs12286929) and HIF1AN (rs17094222) and body fatness for breast cancer risk. Unconditional logistic regression models were applied to measure the associations of related factors with breast cancer by odds ratios (ORs) and 95% confidence intervals (CIs). It was observed that cases had a statistically higher body mass index (BMI ≥ 28 kg/m^2^, OR = 1.77), waist circumference (WC ≥ 90cm, OR = 2.89) and waist-to-hip ratio (WHR ≥ 0.9, OR = 3.41) as compared to controls. Significant differences were also found in the genotype distributions of RASA2 rs16851483 T/T homozygote and CADM1 rs12286929 G/A heterozygote between cases and controls, with an OR of 1.68 (95% CI: 1.10–2.56) and 0.80 (95% CI: 0.64–0.99), respectively. Furthermore, significant interactions were observed between polymorphisms of three genes and body fatness for the risk of breast cancer based on both additive and multiplicative scales. These results of our study suggest that body fatness possibly plays an important role in the development of breast cancer and this risk might be modified by specific genotypes of some potential genes, especially for obese women in China.

## INTRODUCTION

Breast cancer is the most frequently diagnosed cancer and the leading cause of cancer-related death among women globally. It was estimated that 1.7 million cases and 521,900 deaths occurred in 2012, which accounted for 25% of all cancer cases and 15% of all cancer-related deaths among females worldwide [1]. China had the highest number of new breast cancer cases (187,213) among five Asian countries (India 144,937 cases, Japan 55,710 cases, Indonesia 48,998 cases and Pakistan 34,038 cases), accounting for 29% of breast cancer cases in Asian countries [2]. Moreover, the incidence and mortality of this disease in China has risen dramatically in the past years, and this trend will likely continue in the coming years [3].

The development and progression of breast cancer is a multi-step complicated process which involves both environmental and genetic factors [4]. Of the many risk factors identified for breast cancer, body fatness has been considered as an important risk factor especially among postmenopausal women. A strong relationship between body fatness and breast cancer in postmenopausal women has been observed, and it was suggested that an increased body mass index (BMI) was associated with increased incidence of postmenopausal breast cancer [5]. Body fatness might exert its effects based on endogenous hormonal pathways by increasing the production of estrogens through conversion of androstenedione in peripheral adipose tissue [6]. However, the association between breast cancer and body fatness among premenopausal women remains contradictory. In fact, some studies even revealed that high body fatness was a protective factor for premenopausal breast cancer [7–9].

The individual susceptibility of breast cancer is also influenced by genetic factors, including obesity-related genes, such as PPARG, LPL, PON1 and PON2 [10]. Several studies on the association of obesity-related genes with breast cancer have been conducted, but the conclusions were not consistent [11–14]. Recently, genome-wide association studies (GWAS), performed mostly among Caucasians, identified that the alleles of RAS p21 protein activator 2 (RASA2), cell adhesion molecule 1 (CADM1) and hypoxia inducible factor 1 alpha subunit inhibitor (HIF1AN) genes were tightly associated with obesity in the general population [15]. Given the association between body fatness and breast cancer risk, it is biologically plausible that polymorphisms in these genes may be related to the carcinogenesis of breast cancer. Unfortunately, no previous study has been carried out yet. In this study, we performed a large population-based case-control study on breast cancer in China with the purpose of investigating the relationship between RASA2 (rs16851483), CADM1 (rs12286929), HIF1AN (rs17094222) polymorphisms, as well as their joint effects with body fatness and the occurrence of breast cancer among Chinese women.

## RESULTS

The demographic characteristics of participants are presented in Table 1. The average ages of cases and controls were 54.78 ± 11.10 and 54.27 ± 11.30 years old, respectively. Controls had a significantly higher rate of high education level, a higher income in the past 5 years, and a lower rate of first-degree family history of breast cancer and were more often pre-menopausal as compared to cases (*P* < 0.001). There were no statistically significant differences in the distribution of other characteristics between cases and controls.

**Table 1:** Distributions of selected demographic characteristics among cases and controls^a^

Characteristics	Case (*n* = 818) No (%)	Control (*n* = 935) No (%)	*P* value^*^
**Age (years)^b^**			
Mean ± SD	54.78 ± 11.10	54.27 ± 11.30	0.337
< 50	295 (36.1)	328 (35.1)	0.428
50-	239 (29.2)	304 (32.5)	
60-	202 (24.7)	208 (22.3)	
70-	82 (10.0)	95 (10.2)	
**Education levels**			
Primary school/ illiteracy	256 (31.3)	228 (24.4)	< 0.001
Junior high school	348 (42.6)	341 (36.6)	
Middle school & above	213 (26.1)	364 (39.0)	
**Marital status**			
Unmarried	11 (1.4)	9 (1.0)	0.219
Married/living as married	722 (88.4)	848 (90.9)	
Widowed/divorced/separated	84 (10.3)	76 (8.2)	
**Residence area**			
Rural	369 (45.3)	415 (44.8)	0.848
Urban	446 (54.7)	511 (55.2)	
**Per capita income in past five years**			
Mean ± SD	19141.04 ± 22025.98	24831.62 ± 22625.63	< 0.001
<10000	214 (27.2)	143 (15.7)	< 0.001
10000~	284 (36.0)	272 (29.8)	
20000~	290 (36.8)	498 (54.5)	
**First-degree family history of breast cancer**			
No	720 (88.6)	874 (94.4)	< 0.001
Yes	93 (11.4)	52 (5.6)	
**Hormone drugs**			
No	757 (94.5)	884 (96.2)	0.096
Yes	44 (5.5)	35 (3.8)	
**Oral contraceptive**			
No	652 (80.1)	760 (82.2)	0.272
Yes	162 (19.9)	165 (17.8)	
**Age at menarche (years)**			
Mean ± SD	15.64 ± 1.88	15.64 ± 1.90	0.998
<13	28 (3.4)	24 (2.6)	0.298
≥13	784 (96.6)	901 (97.4)	
**Menopausal status**			
Pre-menopause	268 (32.8)	386 (41.3)	< 0.001
Post-menopause	550 (67.2)	549 (58.7)	
**Moderate exercise (hrs/week)**			
0-	185 (23.8)	208 (23.1)	0.165
1-	219 (28.1)	229 (25.4)	
2-	150 (19.3)	213 (23.6)	
3-	225 (28.8)	251 (27.9)	

^a^Missing data were excluded from analysis. ^b^Matching variables. ^*^p-value from the Pearson chi-square test (for categorical variables) and student's *t*-test (for continuous variables) comparing cases and controls.

As shown in Table 2, we found that BMI, WC and WHR had positive associations with the risk of breast cancer. Higher risk was associated with greater BMI, WC and WHR (*P* < 0.001). In detail, compared with the individuals with normal body fatness, the increased risk was observed for overweight (OR = 1.35, 95% CI: 1.08–1.66), obesity (OR = 1.77, 95%CI: 1.29–2.42), WC ≥ 90 (OR = 2.89, 95% CI: 2.17–3.86) and WHR ≥ 0.90 (OR = 3.41, 95% CI: 2.36–4.91). After stratified by menopausal status, this effect was similar in postmenopausal women. For those with BMI ≥ 28 and WC ≥ 90, ORs were 2.19 (95%CI: 1.49–3.23) and 3.56 (95% CI: 2.45–5.18) among postmenopausal women, much higher than those of premenopausal women. WHR was observed to have strong relationship with breast cancer regardless of menopausal status. The adjusted ORs for WHR ≥ 0.90 in premenopausal group and postmenopausal group were 3.27 (95% CI: 1.85–5.79) and 2.42 (95% CI:1.43–4.12), respectively, as compared to individuals with a normal WHR.

**Table 2:** The OR and 95% CI of breast cancer risk with obesity among pre-menopausal and post-menopausal women^a^

Variables	ALL			Premenopause			Postmenopause		
	Case/Ctrl	Model 1	Model 2	Case/Ctrl	Model 1	Model 2	Case/Ctrl	Model 1	Model 2
		OR (95%CI)b	OR (95%CI)c		OR (95%CI)b	OR (95%CI)c		OR (95%CI)b	OR (95%CI)c
**BMI (kg/m2)**									
< 24	344/486	1.00 (referent)	1.00 (referent)	136/224	1.00 (referent)	1.00 (referent)	208/262	1.00 (referent)	1.00 (referent)
24.0–27.9	330/339	1.34 (1.08–1.66)	1.35 (1.08–1.69)	100/125	1.22 (0.85–1.76)	1.17 (0.80–1.72)	230/214	1.39 (1.06–1.82)	1.44 (1.09–1.91)
≥ 28.0	135/110	1.71 (1.27–2.32)	1.77 (1.29–2.42)	30/37	1.33 (0.75–2.34)	1.21 (0.67–2.17)	105/73	2.01 (1.39–2.90)	2.19 (1.49–3.23)
P for trendc		< 0.001	< 0.001		0.120	0.203		< 0.001	< 0.001
**WC (cm)**									
< 80	191/356	1.00 (referent)	1.00 (referent)	105/210	1.00 (referent)	1.00 (referent)	86/146	1.00 (referent)	1.00 (referent)
80-	279/341	1.64 (1.28–2.10)	1.57 (1.21–2.04)	105/121	1.71 (1.17–2.52)	1.49 (1.00–2.22)	174/220	1.51 (1.07–2.14)	1.55 (1.08–2.23)
≥ 90	338/238	2.94 (2.22–3.87)	2.89 (2.17–3.86)	56/55	1.88 (1.16–3.05)	1.74 (1.05–2.87)	282/183	3.48 (2.43–4.97)	3.56 (2.45–5.18)
P for trendc		< 0.001	< 0.001		0.003	0.013		< 0.001	< 0.001
**WHR**									
< 0.80	76/168	1.00 (referent)	1.00 (referent)	43/123	1.00 (referent)	1.00 (referent)	33/45	1.00 (referent)	1.00 (referent)
0.80-	147/255	1.32 (0.93–1.88)	1.35 (0.93–1.95)	71/127	1.51 (0.92–2.48)	1.60 (0.95–2.69)	76/128	0.86 (0.50–1.49)	0.85 (0.48–1.51)
0.85-	193/226	2.05 (1.43–2.92)	2.11 (1.45–3.05)	72/71	2.74 (1.62–4.61)	2.72 (1.57–4.75)	121/155	1.25 (0.74–2.12)	1.32 (0.76–2.28)
≥ 0.90	392/286	3.49 (2.45–4.97)	3.41 (2.36–4.91)	80/65	3.50 (2.02–6.06)	3.27 (1.85–5.79)	312/221	2.49 (1.49–4.16)	2.42 (1.43–4.12)
P for trendc		< 0.001	< 0.001		< 0.001	< 0.001		< 0.001	< 0.001

^a^Missing data were excluded from analysis. ^b^Adjusted for age (continuous), education level, marital status, residence area, previous income (continuous). ^c^Further adjusted for age (continuous), education level, marital status, residence area, previous income (continuous), using of hormone drugs, moderate exercise (continuous) and family history of breast cancer.

The distribution of genotype frequencies for RASA2 (rs16851483), CADM1 (rs12286929) and HIF1AN (rs17094222) polymorphisms between cases and controls, as well as their associations with breast cancer risk are presented in Table 3. After adjusting for potential confounders, the T/T genotype of RASA2 (rs16851483) was associated with an increased risk of breast cancer compared with the G/G genotype (OR = 1.68, 95%CI: 1.10–2.56). Stratifying analyses by menopausal status indicated that RASA2 T/T genotype significantly enhanced breast cancer risk among postmenopausal women (OR = 1.80, 95%CI: 1.05–3.09). CADM1 (rs12286929) G/A genotype was found to be associated with a reduced risk of breast cancer (OR = 0.80, 95%CI: 0.64–0.99), as compared to A/A genotype. Interestingly, a similar result was achieved among premenopausal women instead of postmenopausal group. However, we did not find any significant association between different genotypes of HIF1AN rs17094222 and the occurrence of breast cancer.

**Table 3:** The OR and 95% CI of breast cancer risk with genotype among pre-menopausal and post-menopausal women^a^

Genotype	All			Premenopause			Postmenopause		
	Case/Ctrl	Model 1	Model 2	Case/Ctrl	Model 1	Model 2	Case/Ctrl	Model 1	Model 2
		OR (95% CI)b	OR (95% CI)c		OR (95% CI)b	OR (95% CI)c		OR (95% CI)b	OR (95% CI)c
**RASA2 (rs16851483)**									
G/G	405/502	1.00 (referent)	1.00 (referent)	136/213	1.00 (referent)	1.00 (referent)	269/289	1.00 (referent)	1.00 (referent)
G/T	335/365	1.12 (0.92–1.38)	1.22 (0.98–1.51)	110/142	1.20 (0.85–1.70)	1.24 (0.86–1.80)	225/223	1.06 (0.82–1.37)	1.18 (0.90–1.54)
T/T	58/51	1.54 (1.02–2.31)	1.68 (1.10–2.56)	18/22	1.46 (0.72–2.96)	1.65 (0.80–3.42)	40/29	1.65 (0.98–2.78)	1.80 (1.05–3.09)
*G/T+T/T*	393/416	1.17 (0.96–1.43)	1.27 (1.04–1.56)	128/164	1.23 (0.88–1.73)	1.30 (0.91–1.84)	265/252	1.13 (0.88–1.44)	1.25 (0.96–1.61)
**CADM1 (rs12286929)**									
A/A	442/470	1.00 (referent)	1.00 (referent)	153/186	1.00 (referent)	1.00 (referent)	289/284	1.00 (referent)	1.00 (referent)
G/A	286/381	0.79 (0.64–0.97)	0.80 (0.64–0.99)	92/167	0.70 (0.49–0.99)	0.68 (0.47–0.98)	194/214	0.87 (0.67–1.13)	0.88 (0.67–1.15)
G/G	69/74	1.00 (0.69–1.44)	0.99 (0.67–1.45)	18/27	0.91 (0.47–1.80)	0.83 (0.41–1.68)	51/47	1.07 (0.69–1.68)	1.09 (0.68–1.74)
*G/A+G/G*	355/455	0.82 (0.67–1.00)	0.83 (0.67–1.02)	110/194	0.78 (0.52–1.02)	0.70 (0.49–0.99)	245/261	0.90 (0.71–1.15)	0.91 (0.71–1.18)
**HIF1AN (rs17094222)**									
T/T	345/428	1.00 (referent)	1.00 (referent)	110/182	1.00 (referent)	1.00 (referent)	235/246	1.00 (referent)	1.00 (referent)
T/C	368/381	1.19 (0.96–1.46)	1.17 (0.94–1.46)	122/154	1.35 (0.94–1.94)	1.35 (0.92–1.96)	246/227	1.12 (0.86–1.45)	1.10 (0.84–1.44)
C/C	85/114	0.93 (0.68–1.29)	0.91 (0.65–1.28)	31/43	1.16 (0.68–2.00)	1.27 (0.72–2.25)	54/71	0.83 (0.55–1.26)	0.78 (0.51–1.20)
*T/C+C/C*	453/495	1.13 (0.93–1.37)	1.11 (0.91–1.37)	153/197	1.31 (0.93–1.83)	1.33 (0.93–1.90)	300/298	1.05 (0.82–1.35)	1.02 (0.79–1.33)

^a^Missing data were excluded from analysis. ^b^ Adjusted for age (continuous), education level, marital status, residence area, previous income (continuous). ^c^ Further adjusted for age (continuous), education level, marital status, residence area, previous income (continuous), hormone drugs, moderate exercise (continuous) and first-degree family history of breast cancer.

The joint effects of the aforementioned SNPs and different body fatness indices, including BMI, WC and WHR, on breast cancer risk are shown in Tables 4, 5, 6. Women carrying RASA2 (rs16851483) T/T genotype and BMI ≥ 24 were under a higher risk of breast cancer compared with those who had the G/G genotype and BMI < 24 (OR = 2.51, 95% CI: 1.38–4.56), revealing an additive interaction between specific genotype and BMI. Similarly for WC and WHR, the highest OR was observed among RASA2 T/T carriers with WC ≥ 80 (OR = 3.36, 95% CI: 1.94–5.83) and WHR ≥ 0.85 (OR = 3.50, 95% CI: 1.97–6.25), as compared to G/G genotype carriers with normal WC and WHR, respectively. Compared with CADM1 rs12286929 G/A heterozygotes with BMI < 24 group, individuals with CADM1 A/A+G/G homozygotes and BMI ≥ 24 showed increased risk of breast cancer (OR = 1.78, 95% CI: 1.32–2.40). Similar phenomenon on breast cancer risk was observed for CADM1 polymorphism with WC and WHR. HIF1AN rs17094222 T/C genotype carriers with BMI ≥ 24 had an increased risk compared with the reference group (OR = 1.61, 95% CI: 1.18–2.21). Results from the interactions between WC ≥ 80, WHR ≥ 0.85 and HIF1AN T/C genotypes also showed a significantly increased risk of breast cancer.

**Table 4:** The joint effect between RASA2, CADM1, HIF1AN polymorphisms and BMI on breast cancer^a^

Genotype	BMI < 24 kg/m^2^		BMI ≥ 24 kg/m^2^	
	Case/Ctrl	OR (95% CI)^b^	Case/Ctrl	OR (95% CI)^b^
**RASA2 (rs16851483)**				
G/G	189/258	1.00 (referent)	216/244	1.21 (0.91–1.62)
G/T	128/189	1.00 (0.73–1.38)	207/176	1.70 (1.26–2.30)
T/T	22/29	1.33 (0.72–2.45)	36/22	2.51 (1.38–4.56)
*G/T+T/T*	150/218	1.04 (0.77–1.41)	243/198	1.79 (1.34–2.40)
Interaction	Additive:	RERI = 0.98 (−0.10 to 2.05)		
		AP = 0.44 (0.26–0.62)		
		S = 4.96 (1.54–15.91)		
	Multiplicative:	ROR=1.77 (1.33–2.37)		
**CADM1 (rs12286929)**				
G/A	128/199	1.00 (referent)	158/182	1.31 (0.93–1.82)
A/A	185/249	1.13 (0.82–1.55)	257/221	1.78 (1.31–2.43)
G/G	23/34	1.07 (0.58–1.98)	46/40	1.73 (1.03–2.92)
*A/A+G/G*	208/283	1.12 (0.83–1.53)	303/261	1.78 (1.32–2.40)
Interaction	Additive:	RERI = 1.19 (−0.28 to 2.65)		
		AP = 0.45 (0.25–0.65)		
		S = 3.67 (2.26–5.94)		
	Multiplicative:	ROR=1.77 (1.31–2.39)		
**HIF1AN (rs17094222)**				
T/T	148/221	1.00 (referent)	197/207	1.33 (0.98–1.82)
T/C	163/201	1.14 (0.83–1.57)	205/180	1.61 (1.18–2.21)
C/C	27/58	0.64 (0.37–1.10)	58/56	1.52 (0.96–2.42)
*T/C+C/C*	190/259	1.03 (0.76–1.39)	263/236	1.59 (1.19–2.14)
Interaction	Additive:	RERI = 0.82 (−0.26 to 1.89)		
		AP = 0.37 (0.15–0.59)		
		S = 3.22 (1.79–5.81)		
	Multiplicative:	ROR = 1.58 (1.18–2.13)		

^a^Missing data were excluded from analysis. ^b^Adjusted for age (continuous),education level, marital status, residence area, previous income (continuous), using of hormone drugs, moderate exercise (continuous), first-degree family history of breast cancer and menopausal status.

**Table 5:** Joint effects between RASA2, CADM1 and HIF1AN polymorphisms and WC on breast cancer^a^

Genotype	WC < 80 cm		WC ≥ 80 cm	
	Case/Ctrl	OR (95% CI)^b^	Case/Ctrl	OR (95% CI)^b^
**RASA2 (rs16851483)**				
G/G	102/190	1.00 (referent)	302/312	1.91 (1.38–2.65)
G/T	76/138	1.18 (0.79–1.75)	259/227	2.35 (1.68–3.29)
T/T	11/20	1.41 (0.63–3.16)	47/31	3.36 (1.94–5.83)
*G/T+T/T*	87/158	1.21 (0.82–1.77)	306/258	2.47 (1.78–3.44)
Interaction	Additive:	RERI = 3.48 (−0.61 to7.56)		
		AP = 0.62 (0.44–0.80)		
		S = 4.12 (2.95–5.75)		
	Multiplicative:	ROR = 2.42 (1.74–3.37)		
**CADM1 (rs12286929)**				
G/A	58/149	1.00 (referent)	227/232	2.68 (1.81–3.97)
A/A	117/175	1.81 (1.20–2.73)	325/295	2.94 (2.01–4.29)
G/G	13/27	1.40 (0.66–3.00)	56/47	3.22 (1.86–5.56)
*A/A+G/G*	130/202	1.75 (1.17–2.62)	381/342	2.97 (2.05–4.32)
Interaction	Additive:	RERI = 9.84 (−2.57 to 22.26)		
		AP = 0.75 (0.59–0.90)		
		S = 5.18 (3.13–8.58)		
	Multiplicative:	ROR = 2.90 (2.00–4.22)		
**HIF1AN (rs17094222)**				
T/T	79/163	1.00 (referent)	266/265	1.95 (1.37–2.76)
T/C	92/143	1.17 (0.78–1.75)	275/238	2.30 (1.62–3.26)
C/C	17/43	0.85 (0.44–1.62)	68/71	1.83 (1.14–2.92)
*T/C+C/C*	109/186	1.09 (0.74–1.61)	343/309	2.19 (1.56–3.08)
Interaction	Additive:	RERI = 2.43 (−0.81 to5.67)		
		AP = 0.55 (0.33–0.77)		
		S = 3.47 (2.44–4.94)		
	Multiplicative:	ROR=2.14 (1.52–3.01)		

^a^ Missing data were excluded from analysis.^b^Adjusted for age (continuous), education level, marital status, residence area, previous income (continuous), using of hormone drugs, moderate exercise (continuous), first-degree family history of breast cancer and menopausal status.

**Table 6:** Joint effects between RASA2, CADM1 and HIF1AN polymorphisms and WHR on breast cancer^a^

Genotype	WHR < 0.85		WHR ≥ 0.85	
	Case/Ctrl	OR (95% CI)^b^	Case/Ctrl	OR (95% CI)^b^
**RASA2 (rs16851483)**				
G/G	117/232	1.00 (referent)	288/270	2.23 (1.63–3.06)
G/T	86/159	1.15 (0.79–1.67)	249/206	2.69 (1.94–3.72)
T/T	18/24	1.94 (0.98–3.83)	40/27	3.50 (1.97–6.25)
*G/T+T/T*	104/183	1.25 (0.88–1.78)	289/233	2.79 (2.03–3.82)
Interaction	Additive:	RERI = 5.03 (−0.26 to 10.31)		
		AP = 0.67 (0.52–0.82)		
		S = 4.47 (3.12–6.40)		
	Multiplicative:	ROR = 2.72 (1.98–3.74)		
**CADM1 (rs12286929)**				
G/A	75/184	1.00 (referent)	211/197	2.88 (1.99–4.15)
A/A	133/203	1.69 (1.17–2.45)	309/267	2.99 (2.11–4.24)
G/G	12/31	1.00 (0.46–2.16)	57/43	3.54 (2.09–6.99)
*A/A+G/G*	145/234	1.60 (1.11–2.30)	366/310	3.06 (2.18–4.31)
Interaction	Additive:	RERI = 9.96 (−1.30 to 21.21)		
		AP = 0.75 (0.61–0.88)		
		S = 5.16 (3.26–8.18)		
	Multiplicative:	ROR = 3.00 (2.13–4.22)		
**HIF1AN (rs17094222)**				
T/T	88/190	1.00 (referent)	257/238	2.34 (1.66–3.29)
T/C	105/182	1.15 (0.79–1.67)	263/199	2.88 (2.03–4.08)
C/C	27/44	1.33 (0.75–2.37)	58/70	1.75 (1.09–2.80)
*T/C+C/C*	132/226	1.18 (0.83–1.69)	321/269	2.58 (1.87–3.61)
Interaction	Additive:	RERI = 4.05 (−0.75 to8.84)		
		AP = 0.63 (0.45–0.81)		
		S = 3.93 (2.67–5.80)		
	Multiplicative:	ROR = 2.51 (1.79–3.51)		

^a^ Missing data were excluded from analysis.^b^Adjusted for age (continuous), education level, marital status, residence area, previous income (continuous), using of hormone drugs, moderate exercise (continuous), first- degree family history of breast cancer and menopausal status.

## DISCUSSION

To the best of our knowledge, this study is the first to report the effects of rs16851483 (RASA2), rs12286929 (CADM1) and rs17094222 (HIF1AN) polymorphisms and their interactions with high body fatness on the development of breast cancer among Chinese women. Results showed that the T/T genotype of RASA2 (rs16851483) was associated with increased breast cancer risk, and the G/A genotype of CADM1 (rs12286929) might play a role in reducing the risk of breast cancer. Moreover, we found that high body fatness such as BMI ≥ 28kg/m^2^, WC ≥ 90cm and WHR ≥ 0.9 significantly enhanced the risk of breast cancer, which could be modified by the polymorphisms of several potential genes.

Etiologic evidence indicates that breast cancer is a complicated disease which is affected by environmental, genetic, and economic conditions as well as lifestyles [16]. Body fatness has been suggested as an important risk factor resulting in breast cancer. A meta-analysis among Chinese women showed that the OR (95%CI) of obesity was 1.08 (1.04–1.12) as compared to normal BMI, indicating that obesity was a risk factor for breast cancer [17]. Prospective cohort study among Chinese women also revealed that both general obesity and central obesity easily suffered from several cancers, including breast cancer [18]. Interestingly, this association might be affected by menopausal status. It has been reported that obesity reduced risk of breast cancer in premenopausal women, whereas it was associated with an increased risk in postmenopausal women [19, 20]. The pre- and postmenopausal change in risk is likely related to estrogen production. Premenopausal estrogen is secreted by the ovary, while after menopause, estrogen is mainly produced by fat [21]. However, the exact mechanism is still unknown. In this large population-based study, obese women (BMI ≥ 28kg/m^2^) were observed to have an increased risk of developing breast cancer, especially in postmenopausal women. Furthermore, postmenopausal women with greater WC or WHR had a higher risk of breast cancer as compared to normal individuals. Our findings were consistent with those from several previous studies [22, 23]. As the burden of obesity in the world is increasing, particularly in China [24], it could be expected that the incidence of breast cancer will continue to rise among Chinese women.

Obesity could be affected by multiple genes [25]. The mechanism by which obesity-related genes influence obesity in humans remains unclear. A possible mechanism is that obesity-related genes play a vital role in energy homeostasis, metabolism and adipogenesis [26]. Studies have suggested that variations in obesity-related genes could alter the level of circulating ghrelin to add weight gain mainly by choosing energy dense foods and diminishing the sensation of satiety [27, 28]. In addition, obesity-related SNPs might also be involved in breast carcinogenesis. In this study, we investigated the association of three polymorphic sites, RASA2 (rs16851483), CADM1 (rs12286929) and HIF1AN (rs17094222), with breast cancer risk among Chinese women based on previously identified obesity-related SNPs from the GWAS study [15].

The SNP rs16851483 is located in intron 7 of RASA2 gene. The protein encoded by RASA2 gene is a member of the GAP1 family of GTPase-activating proteins and stimulates GTPase activity of normal RAS p21 (wild-type RAS), but not its oncogenic counterpart. Acting as a suppressor of RAS function, RASA2 protein enhances weak intrinsic GTPase activity of RAS proteins to result in the inactive GDP-bound form of RAS, thus allowing control of cellular proliferation, survival, differentiation, motility and transcription [29, 30]. RASA2 is a potential tumor-suppressor gene. Its variation or loss will alleviate RAS suppression and may therefore lead to carcinogenesis. Moreover, the variation in the abundance of endogenous RASA2 protein among different cell lines could modulate RAS complex formation, contributing to context-specific signaling plasticity [31]. In some cases, the RASA2 mutants were also found to enhance RAS-GTP levels, indicating that the underlying variants might have dominant-negative effects [32]. Our results showed that the mutant T/T genotype of RASA2 (rs16851483) was associated with an increased risk of breast cancer, and this finding was more pronounced in postmenopausal women. Although previous studies demonstrated RASA2 inactivation as a driver of several cancers including melanoma and Sézary Syndrome [33, 34], no published studies have reported its association with breast cancer. Whether this polymorphism of RASA2 gene influenced intracellular protein function to promote the development of breast cancer depends on further investigation in the near future. Meanwhile, we also observed significant interaction between RASA2 genotypes and high body fatness for breast cancer susceptibility, suggesting that the specific genotype of RASA2 could modify the risk of breast cancer induced by high body fatness.

The SNP rs12286929 is located in the 5’-noncoding region of CADM1 gene. CADM1, also known as TSLC1 (Tumor Suppressor in Lung Cancer 1), was first found in human chromosome 11q23.2 as a new suppressor gene when exploring correlation between human non-small cell lung cancer and CADM1 through functional experiments [35]. The immunoglobulin superfamily molecule encoded by CADM1 is involved in cell–cell adhesion in a variety of human epithelia, including mammary glands [36]. Promoter methylation of CADM1 has been shown to be one of the main mechanisms to drive the initiation and progression of various cancers [35, 37]. However, loss of heterozygosity (LOH) at the chromosomal region in which CADM1 is located, would act as a “second hit” to inactivate the gene beyond the effect of promoter methylation [38, 39]. Wikman et al also found that loss of CADM1 expression was associated with poor prognosis and brain metastasis in breast cancer patients [40]. In this study, we uncovered that the G/A heterozygotes of CADM1 (rs12286929) were associated with a decreased risk of breast cancer. Considering the possible distinct molecular pathways underlying tumorigenesis depending on menopausal status, stratified analyses were conducted and the obtained results showed that the association only existed in premenopausal breast cancer. Significant interaction between CADM1 polymorphism and high body fatness on breast cancer was observed in our study. Further studies are needed to explore the definite mechanism for the effect of this interaction on breast cancer risk.

The SNP rs17094222 is located in 3’ untranslated region of HIF1AN gene. HIF1AN, also called FIH-1 (factor-inhibiting hypoxia inducible factor 1), is an asparaginyl β-hydroxylase enzyme to hydroxylate the HIF-α, thus preventing its transcriptional activity in response to hypoxia [41]. HIF-1 is a transcription factor that regulates oxygen homeostasis and plays a key role in the development of diseases [42]. For instance, the expression and distribution of HIF-1α were found to be associated with human cancer progression in bladder, brain, kidney, breast, ovary, pancreas and prostate [43–45]. Additionally, HIF-1α displays two expression patterns: a canonical hypoxia-related perinecrotic pattern and a diffuse expression pattern, both related to a poor clinical outcome in breast cancer [46]. As an important factor regulating HIF-1α transcriptional activity, the role of HIF1AN in breast carcinogenesis could be rationally assumed. Zhang et al evaluated the association of several SNPs in HIF1AN with acute mountain sickness [47]. However, the variants in HIF1AN for the susceptibility to breast cancer have not been previously assessed in detail. In this study, although the positive association between the genotypes of rs17094222 and breast cancer risk was not obtained, the joint effect of BMI ≥ 24 and HIF1AN T/C heterozygote was observed to increase the risk to develop breast cancer. Similar effects were also found when WC ≥ 80 and WHR ≥ 0.85 were combined with different genotypes of HIF1AN rs17094222 (T/T, T/C, C/C and T/C+C/C). These results might attribute to HIF1AN gene silence caused by high body fatness, while HIF1AN activity is essential for preventing tumor growth [48].

This study provided a novel insight into the roles of obesity and gene polymorphisms of RASA2, CADM1 and HIF1AN in the development of breast cancer. However, there were several limitations in the present study. First, body fatness indices including BMI, WC and WHR were calculated based on measurement after disease occurrence. It is possible that lifestyle changes post cancer diagnosis might influence body fatness. Actually we tried to collect such information before disease onset, however, almost 50% of data were missing because cases were not able to remember their accurate information before disease onset. Second, the selected obesity-related gene polymorphisms were initially identified in western countries and have not been sufficiently studied among Chinese population. More evidence is required to support the findings from the present study. Third, we did not obtain the information on estrogen receptor (ER) status of breast cancer. So the association was not analyzed according to ER positive and ER negative tumors.

In conclusion, our study confirms that body fatness plays an important role in the development of breast cancer in Chinese population, especially among postmenopausal women. Such risk might be modified by specific polymorphisms of obesity-related genes such as RASA2 rs16851483, CADM1 rs12286929 and HIF1AN rs17094222, yielding some novel insights into breast carcinogenesis. Genetic predispositions together with body fatness and other lifestyle factors may ultimately determine individual risk of breast cancer in Chinese women. These meaningful findings can be potentially used to evaluate individual inherited susceptibility to breast cancer, especially for those women with overweight and obesity, thus screening high risk persons in public health, as well as provide effective biological markers for early diagnosis of breast cancer in the clinic. It must be also noted that much more work still requires to be done based on larger cohort studies with different ethnical backgrounds in the future to further validate our current results.

## MATERIALS AND METHODS

### Ethics statement

This study was performed according to the Declaration of Helsinki and approved by the Ethics Committee of Jiangsu Provincial Center for Disease Control and Prevention (Nanjing, China). The written informed consent was obtained from all participants.

### Study design

A population-based case-control study on breast cancer has been conducted in Wuxi City of Jiangsu Province from 2013–2014. Wuxi is a developed city located in the south of Jiangsu, with a total population of 4,802,673 in 2015. Women accounted for nearly 50% of the population.

All study participants were restricted to local female residents who have lived in the study area for at least 5 years. Cases consisted of newly diagnosed primary breast cancer patients, and information from the local population-based cancer registry was used to exclude recurrent patients. All cases were identified according to the International Classification of Diseases, tenth revision (ICD-10, code C50). Eligible controls were randomly selected from the general population and were derived from the same area as cases, frequency-matched within 5 years of age. Individuals with any history of cancer were excluded. During the study period, a total of 1,410 new breast cancer cases were identified in local inhabitants and 1,072 controls were selected. Finally, 864 cases and 940 controls were recruited. The corresponding response rates of cases and controls were 61.3% and 87.7%, respectively. After excluding participants who had incomplete information, 818 cases and 935 controls were included in data analysis.

All eligible participants were invited to Wuxi Maternal and Child Health Hospital, which is the largest women's and children's healthcare hospital in Wuxi City. With written informed consent, a pre-tested structured questionnaire was used to obtain epidemiological data by trained investigators through face-to-face interviews. The questionnaire included detailed information on known or potential risk or protective factors for breast cancer, such as demographic information, socio-economic status, moderate physical activity, disease history, etc. Anthropometric measurements including height, weight, waist circumference, and hip circumference were obtained at the time of interview. Because most cases couldn't remember accurate body fatness before disease occurrence, values of on-site measurements were used in subsequent analysis. Blood samples were collected by 5ml EDTA anticoagulation tube for further lab analyses.

### SNP selection, DNA isolation and genotyping analyses

The newly identified 20 genomic loci most tightly associated with obesity from previous genetic study [15] were initially selected to investigate the relationship with breast cancer. Preliminary experimental data excluded 17 SNPs because they had not the associations with breast cancer risk or significant interactions with body fatness indices. Therefore, the remaining 3 SNPs, 3q23-rs16851483 (RASA2), 11q23.3-rs12286929 (CADM1) and 10q24.3-rs17094222 (HIF1AN), were chosen for detailed analysis in the subsequent study.

DNA was extracted from 200 μl of peripheral blood using QIAamp DNA Blood Mini Kit (QIAGEN, Germany) following the manufacturer's instructions. All extracted DNA was stored at −80°C. RASA2 rs16851483, CADM1 rs12286929 and HIF1AN rs17094222 genotyping among cases and controls were performed using the Sequenom MassARRAY platform provided by CapitalBio (Beijing, China). Around 5% of randomly selected samples were repeated for quality control. Call rates were above 95% and concordance was observed at 100%. Hardy-Weinberg equilibrium (HWE) was assessed for defined different genotypes. All frequencies of these three SNPs conformed to Hardy-Weinberg equilibrium among controls (*P* > 0.05).

### Statistical analyses

In present study, body fatness indices included body mass index (BMI), waist circumference (WC) and waist-to-hip ratio (WHR). BMI is a measure of overall adiposity, while WC and WHR are commonly used to measure abdominal or central adiposity. BMI was calculated as weight in kilograms (kg) divided by height in squared meters. WHR was calculated by using WC in centimeters (cm) divided by hip circumference in cm. The cutoff points of BMI were defined according to China Adults Overweight and Obesity Prevention and Control Guidelines: BMI < 18.5 kg/m^2^ as underweight, 18.5 kg/m^2^ ≤ BMI < 24.0 kg/m^2^ as normal, 24.0 kg/m^2^ ≤ BMI < 28.0 kg/m^2^ as overweight and BMI ≥ 28.0 kg/m^2^ as obesity. Due to the low number of women falling into the underweight category in our population, we integrated it into the normal group to form three categories of BMI for data analysis. We defined WC ≥ 80 cm and WHR ≥0.85 as an increase according to the recommendation of the World Health Organization [49–51].

Pearson's Chi-square test (categorical variables) and Student's *t*-test (continuous variables) were used to compare the differences in demographic characteristics between cases and controls. Unconditional logistic regression models were applied for evaluating the main associations of different genotypes and body fatness, as well as potential gene-environment interactions with odds ratios (ORs) and 95% confidence intervals (CIs). Confounders were selected based on the results of our preliminary data analysis and previous studies on breast cancer [52], including age (continuous), education level (categorical), marital status (categorical), residence area (categorical), previous income (continuous), hormone drugs use (categorical), family history of breast cancer (categorical), and moderate exercise (continuous). Model 1 was adjusted basic stable socio-economic indicators (age, education level, marital status, residence area, previous income) and model 2 was further adjusted some confounders that had greater impact on outcome of breast cancer (hormone drugs use, family history of breast cancer, and moderate exercise). Stratified analyses by menopausal status were used to evaluate potential effect modification.

Statistical interactions between BMI, WC, WHR and genotypes on breast cancer risk were assessed based on both additive and multiplicative scales. BMI < 24 kg/m^2^, WC < 80 cm and WHR < 0.85 with lowest risk genotype were defined as the reference group. The multiplicative interaction was assessed by including the main effect variables and their product terms in the logistic regression model. For the additive interaction, three measures: relative excess risk due to interaction (RERI), attributable proportion due to interaction (AP), and synergy index (SI) were calculated [53]. The 95% CIs of RERI, SI, and AP were estimated by the delta method [54, 55]. In the absence of an interaction, RERI and AP amount to 0, while SI amounts to 1.

Data were analyzed using SAS Office Analytics Server 9.4 (SAS Institute Inc., Cary, NC, USA.).
